# Effectiveness of Electroencephalography Neurofeedback for Improving Working Memory and Episodic Memory in the Elderly: A Meta-Analysis

**DOI:** 10.3390/medicina60030369

**Published:** 2024-02-22

**Authors:** Yu-Ru Lin, Tien-Wei Hsu, Che-Wei Hsu, Peng-Yu Chen, Ping-Tao Tseng, Chih-Sung Liang

**Affiliations:** 1 Graduate Institute of Psychology, College of Humanities and Social Science, Kaohsiung Medical University, Kaohsiung 807, Taiwan; 2 Department of Psychiatry, E-DA Dachang Hospital, I-Shou University, Kaohsiung 807, Taiwan; 3 Department of Psychiatry, E-DA Hospital, I-Shou University, Kaohsiung 807, Taiwan; 4 Graduate Institute of Clinical Medicine, College of Medicine, Kaohsiung Medical University, Kaohsiung 807, Taiwan; 5 Department of Psychology, Kaohsiung Kai-Suan Psychiatric Hospital, Kaohsiung 807, Taiwan; hsu1219@livemail.tw; 6 Department of Psychology, Pingtung Veterans Hospital, Pingtung 900, Taiwan; q879@ptvgh.gov.tw; 7 Institute of Biomedical Sciences, National Sun Yat-sen University, Kaohsiung 807, Taiwan; 8 Department of Psychology, College of Medical and Health Science, Asia University, Taichung 413, Taiwan; 9 Prospect Clinic for Otorhinolaryngology & Neurology, Kaohsiung 807, Taiwan; 10 Institute of Precision Medicine, National Sun Yat-sen University, Kaohsiung 807, Taiwan; 11 Department of Psychiatry, Tri-Service General Hospital, Beitou Branch, Taipei 114, Taiwan; 12 Department of Psychiatry, National Defense Medical Centre, Taipei 114, Taiwan

**Keywords:** neurofeedback, memory function, elderly

## Abstract

*Background and Objective*: Existing evidence indicates the potential benefits of electroencephalography neurofeedback (NFB) training for cognitive function. This study aims to comprehensively review all available evidence investigating the effectiveness of NFB on working memory (WM) and episodic memory (EM) in the elderly population. *Material and Methods*: A systematic search was conducted across five databases to identify clinical trials examining the impact of NFB on memory function in healthy elderly individuals or those with mild cognitive impairment (MCI). The co-primary outcomes focused on changes in WM and EM. Data synthesis was performed using a random-effects meta-analysis. *Results*: Fourteen clinical trials (*n* = 284) were included in the analysis. The findings revealed that NFB was associated with improved WM (k = 11, reported as Hedges’ g = 0.665, 95% confidence [CI] = 0.473 to 0.858, *p* < 0.001) and EM (k = 12, 0.595, 0.333 to 0.856, *p* < 0.001) in the elderly, with moderate effect sizes. Subgroup analyses demonstrated that NFB had a positive impact on both WM and EM, not only in the healthy population (WM: k = 7, 0.495, 0.213 to 0.778, *p* = 0.001; EM: k = 6, 0.729, 0.483 to 0.976, *p* < 0.001) but also in those with MCI (WM: k = 6, 0.812, 0.549 to 1.074, *p* < 0.001; EM: k = 6, 0.503, 0.088 to 0.919, *p* = 0.018). Additionally, sufficient training time (totaling more than 300 min) was associated with a significant improvement in WM (k = 6, 0.743, 0.510 to 0.976, *p* < 0.001) and EM (k = 7, 0.516, 0.156 to 0.876, *p* = 0.005); however, such benefits were not observed in groups with inadequate training time. *Conclusions*: The results suggest that NFB is associated with enhancement of both WM and EM in both healthy and MCI elderly individuals, particularly when adequate training time (exceeding 300 min) is provided. These findings underscore the potential of NFB in dementia prevention or rehabilitation.

## 1. Introduction

Memory function decline in the elderly is a natural and common aspect of aging, often characterized by alterations in cognitive abilities related to the encoding, storage, and retrieval of information [1]. While mild memory decline is considered a normal part of aging, more severe and persistent cognitive impairment may indicate conditions like mild cognitive impairment (MCI) or even neurodegenerative diseases such as Alzheimer’s disease [1]. As individuals age, the dynamic nature of memory undergoes diverse changes, exerting distinct influences on cognitive abilities.

Neurofeedback (NFB) represents a sophisticated and non-invasive form of brain training, grounded in the principles of operant conditioning [2]. NFB involves real-time monitoring of the brain’s electrical activity, typically through the use of electroencephalography (EEG), with subsequent feedback provided to individuals in an effort to modulate the function of brain regions and/or rhythms of interest [3]. During NFB training, different EEG rhythms can be directed for different purposes. In EEG studies, compared to healthy controls, patients with Alzheimer’s disease were suggested to have a decreased power in higher frequencies (alpha, 8–12 Hz and beta, 15–30 Hz) [4,5]. In healthy participants, alpha and beta bands may also reflect individual memory capacity [6]. Theta oscillations (4–8 Hz) also play a role in encoding episodic memories and are correlated with behavioral performance [7]. A quantitative EEG study also suggested that increased relative theta power was significantly correlated with cognitive function and may be the first change in patients with Alzheimer’s disease [8]. In healthy populations, meta-analytic studies have suggested that alpha NFB and theta NFB might improve working memory and episodic memory [9,10]. NFB has been applied to memory function in patients with MCI, and a positive effect on memory enhancement was reported [11,12,13]. A clinical trial reported a positive effect of theta-down NFB on learning memory and overall cognitive performance in patients with Alzheimer’s disease [14]. Several systemic reviews provided evidence of NFB in the elderly [15,16,17]. Jiang et al. [15] reviewed 13 clinical trials of NFB on working memory function in elderly people (including healthy, MCI, and stroke), and 11 trials reported positive findings. Laborda–Sánchez et al. [16] reviewed 14 clinical trials of NFB on aging-associated cognitive decline, and they reported that NFB improved memory in healthy and unhealthy participants, mainly when the theta waves and SMR were trained. Conversely, NFB had no effect on attention processes. Trambaiolli et al. [17] reviewed 10 studies of NFB in patients with cognitive function impairment (6 for MCI and 4 for dementia) and found that most studies reported a positive effect on cognitive functions. However, the studies included in these systematic reviews had varying populations and differences in outcome measurements. Currently, there is no meta-analysis to provide integrated evidence.

To fill this gap, the present systematic review and meta-analysis aimed to investigate the effectiveness of NFB on memory function, including working memory and episodic memory, in the elderly population without major clinical conditions. We also examined the effectiveness of NFB between healthy elderly individuals and those with MCI, as well as the optimal training parameters for specific subpopulations. We chose to include MCI because, compared to dementia, individuals with MCI are still able to maintain independence and perform daily tasks, with cognitive decline being relatively mild. In addition, both healthy elderly individuals and those with MCI are non-clinical individuals residing in the community. We anticipate that NFB could be applied for the prevention and rehabilitation of cognitive function in community-dwelling elderly individuals.

## 2. Materials and Methods

The protocol of the current study was pre-registered a priori with the Open Science Framework (OSF) (https://osf.io/q94k5/, accessed on 9 February 2024). This meta-analysis adheres to the reporting guidelines outlined in the Preferred Reporting Items for Systematic Reviews and Meta-analyses (PRISMA) 2020 [18] (Supplement S1).

### 2.1. Search Strategy

The Cochrane Central Register of Controlled Trials (CENTRAL), PubMed, EMBASE, Web of Science, and ClinicalTrials.gov were systematically searched. The search terms were as follows: (EEG OR electroencephalograph*) AND (neurofeedback OR biofeedback) AND (memor* OR cogniti*) AND (elderly OR old OR older OR MCI OR mild cognitive impairment), with various filters applied in different search platforms. Details of the search strategy can be found in Supplement S2, and the reasons for exclusion are outlined in Supplement S3.

The PICOS settings of the current meta-analysis are as follows: (P) elderly, with or without MCI, (I) EEG neurofeedback, (C) no neurofeedback, sham control, (O) working memory and episodic memory, and (S) clinical trials. Screening and selection of studies were independently conducted by four authors, with each study assessed by a minimum of two authors. Disagreements were resolved through consultation with the corresponding author.

### 2.2. Inclusion and Exclusion Criteria

The screening and selection of studies were performed independently by two authors; each study was assessed by a minimum of two authors. Disagreements were resolved by consulting with the corresponding author. Clinical trials were included, both parallel clinical trials and single-arm pre–posttrials. In cases of head-to-head clinical trials, the NFB arm was seen as a pre–post arm. Our study is focused on community-dwelling elderly individuals who are capable of living independently. Therefore, the studies used meet the following criteria: (1) mean age > 60 years; (2) no major cognitive disability (i.e., individuals with MCI were eligible); (3) the brain regions (e.g., frontal lobe or EEG location, P3/P4) undergoing training and the specific EEG rhythm (e.g., 12–15 Hz, or theta rhythm) being trained need to be clearly defined in the study in order to precisely understand the treatment protocol; (4) use of structured memory paradigms or neuropsychological tests. We did not restrict the educational level, measurement tools, intervention sessions, and intervention duration for a boarder review of studies. Working memory is defined as the ability to temporarily store and manipulate information. Episodic memory is defined as the ability to encode, consolidate, and retrieve experiences. We excluded studies recruiting patients with stroke, traumatic brain injury, brain metastasis, substance use disorder, any types of dementia, or major psychiatric diseases (e.g., major depressive disorder, bipolar disorder, or schizophrenia).

### 2.3. EEG Band Definition

The following were coded: theta (4–8 Hz), alpha (8–12 Hz), beta (12–30 Hz), which includes both the sensory-motor rhythm (12–15 Hz or 12–18 Hz), and gamma (30–100 Hz).

### 2.4. Data Extraction and Outcome Definition

The co-primary outcomes were the change in working memory and episodic memory function scores at the end of NFB. We extracted baseline, post-treatment, and change in scores for memory (means and standard deviations). If two measures met the criteria, we extracted the measure with the lower *p*-value. For each study, we also extracted the following data: (1) trial characteristics (e.g., sample size, authors, country, number of sessions, duration of sessions, electrode positions, neurofeedback modality) and patient characteristics (e.g., age, sex, healthy elderly or elderly with MCI).

### 2.5. Assessment of Bias

Two independent reviewers assessed each study for bias using JADAD quality scores [19]. The JADAD scale is a three-point questionnaire used to assess the methodological quality of clinical trials. It focuses on three key features: randomization, blinding, and a description of withdrawals and dropouts. There are seven questions in total, with one point given for a positive answer to five questions and one point subtracted for a positive answer to the other two questions. The total score ranges from zero (very poor) to five (rigorous) for evaluating the methodological quality of the clinical trial.

### 2.6. Data Analysis

A restricted maximum-likelihood random-effects model was used to calculate the effect size (Hedges’ g statistic) with 95% confidence intervals (CIs). An intention-to-treat approach was used in this study. For the interpretation of effect sizes, we followed the guidelines of classifying <0.2 as very small, 0.2–0.5 as small, 0.5–0.8 as moderate, and >0.8 as large. The I2 statistic was used to quantify heterogeneity across studies, with values of 25%, 50%, and 75% reflecting low, medium, and high degrees of heterogeneity, respectively. The subgroup analyses included MCI versus healthy controls, neurofeedback modality, treatment duration, and trial design. Subgroup meta-analysis was performed when at least three datasets were available. Meta-regression analyses examined the following variables: mean age, education level, and treatment duration. Data management and analysis were carried out using Comprehensive Meta-Analysis software, version 3 (Biostat, Englewood, NJ, USA). For studies that reported effect estimates graphically, a web plot digitizer (www://plotdigitizer.sourceforge.net/, accessed on 9 February 2024) was used to estimate the effect estimates from the graphs.

## 3. Results

### 3.1. Study Characteristics

**Figure 1:**
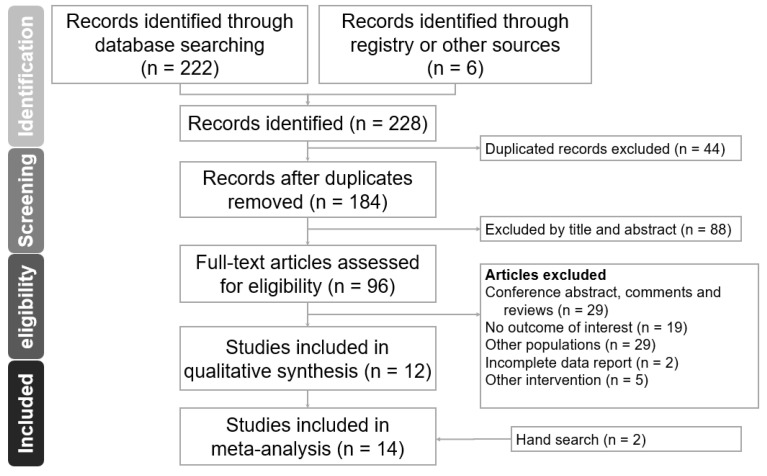
Flow chart of search strategy.

We included 14 clinical trials with 284 participants (186 in the NFB group and 98 in the control group) with ages ranging from 64.6 to 79.2 years (Figure 1, Table 1). Six studies recruited elderly individuals with MCI [11,12,13,20,21,22], while another eight studies included healthy elderly individuals [23,24,25,26,27,28,29,30]. There were eleven controlled trials (three blinded and eight open-label), and three were open-label pre–post design.

### 3.2. Methodology Quality of the Included Studies

The quality of the included studies was evaluated using the JADAD scores, and the results are summarized in Supplement S4. Among the 14 studies we included, only 1 study, which adopted a double-blind randomized controlled trial (RCT) design, received 4 points on the JADAD scale [29]. All other studies employed a single-blind or open-label approach, so scores for blinding criteria could not be obtained. Three studies were open-label pre–post studies [13,21,22], and one was a non-randomized controlled trial [24]. These four studies could not provide scores for randomization criteria and, therefore, received 1 point on the JADAD scale. The other nine studies employed a randomized controlled approach and received 2 points on the JADAD scale.

### 3.3. Co-Primary Outcomes: Working Memory and Episodic Memory

**Figure 2:**
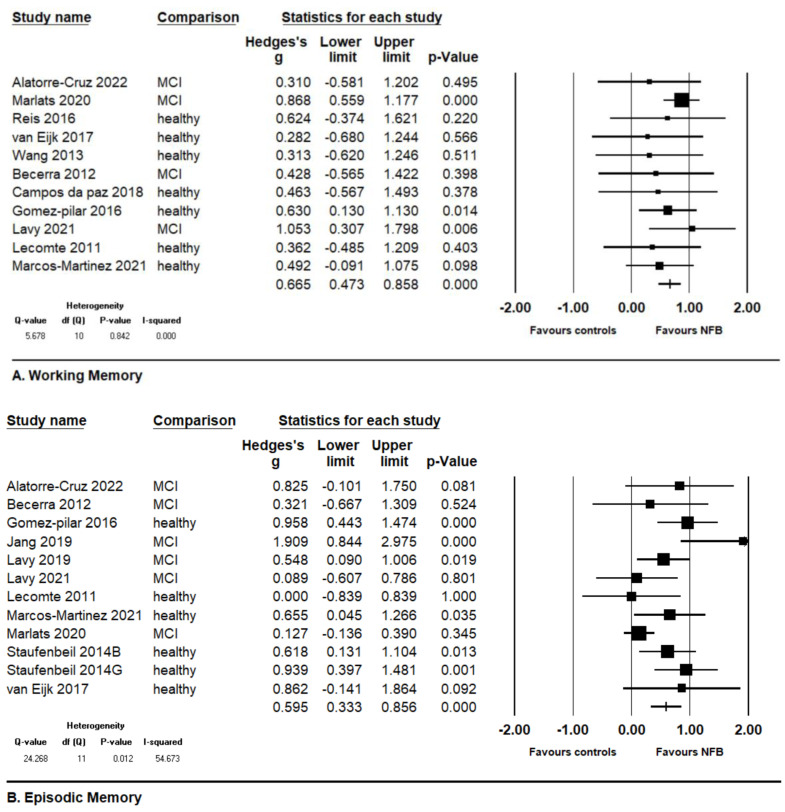
Forest plot of meta-analysis of change of (**A**) working memory and (**B**) episodic memory in elderly people receiving neurofeedback training [11,12,13,20,21,22,23,24,25,26,27,28,29,30].

When all studies were pooled together, NFB significantly improved working memory function in the elderly without significant heterogeneity (k = 11, Hedges’ g = 0.665, 95% confidence interval [CI] = 0.473 to 0.858, *p*-value < 0.001, I^2^ = 0, Figure 2A). NFB also significantly improved episodic memory function in the elderly, but with moderate heterogeneity (k = 12, Hedges’ g = 0.595, 95% CI = 0.333 to 0.856, *p*-value < 0.001, I^2^ = 54.673, Figure 2B).

### 3.4. Subgroup Analyses

The results of subgroup analyses are presented in Table 2.

#### 3.4.1. Controlled Studies vs. Single-Arm Pre–Post Studies

Both controlled studies (k = 9, Hedges’ g = 0.548, 95% CI = 0.277 to 0.818, *p*-value < 0.001, I^2^ = 0) and pre–post studies (k = 2, Hedges’ g = 0.765, 95% CI = 0.436 to 1.093, *p*-value < 0.001, I^2^ = 19.662) demonstrated a working memory enhancement effect without significant heterogeneity. Both controlled studies (k = 6, Hedges’ g = 0.539, 95% CI = 0.170 to 0.909, *p*-value = 0.004, I^2^ = 24.664) and pre–post studies (k = 6, Hedges’ g = 0.662, 95% CI = 0.282 to 1.042, *p*-value = 0.001, I^2^ = 70.938) demonstrated an episodic memory enhancement effect, but the pre–post studies group had moderate heterogeneity.

#### 3.4.2. Healthy Elderly vs. Elderly with MCI

NFB demonstrated working memory (k = 6, Hedges’ g = 0.812, 95% CI = 0.549 to 1.074, *p*-value < 0.001, I^2^ = 0) and episodic memory (k = 6, Hedges’ g = 0.503, 95% CI = 0.088 to 0.919, *p*-value = 0.018, I^2^ = 61.208) enhancement effects in elderly people with MCI. In healthy elderly people, NFB also demonstrated working memory (k = 7, Hedges’ g = 0.495, 95% CI = 0.213 to 0.778, *p*-value = 0.001, I^2^ = 0) and episodic memory (k = 6, Hedges’ g = 0.729, 95% CI = 0.483 to 0.976, *p*-value < 0.001, I^2^ = 0) enhancement effects.

#### 3.4.3. More Than 10 Sessions vs. Less Than 10 Sessions

Regarding the number of training sessions, both more than 10 sessions (working memory, k = 7, Hedges’ g = 0.728, 95% CI = 0.491 to 0.966, *p*-value < 0.001, I^2^ = 0; episodic memory, k = 7, Hedges’ g = 0.534, 95% CI = 0.148 to 0.920, *p*-value = 0.007, I^2^ = 57.847) of training and less than 10 sessions of training improved working memory and episodic memory in the elderly (working memory, k = 4, Hedges’ g = 0.546, 95% CI = 0.219 to 0.873, *p*-value = 0.001, I^2^ = 0; episodic memory, k = 5, Hedges’ g = 0.716, 95% CI = 0.445 to 0.986, *p*-value < 0.001, I^2^ = 10.813).

#### 3.4.4. More Than 300 Min Total Training Time vs. Less Than 300 Min

When NFB training time was more than 300 min, NFB significantly improved both working memory (k = 6, Hedges’ g = 0.743, 95% CI = 0.510 to 0.976, *p*-value < 0.001, I^2^ = 0) and episodic memory (k = 7, Hedges’ g = 0.516, 95% CI = 0.156 to 0.876, *p*-value = 0.005, I^2^ = 57.381). When NFB training time was less than 300 min, NFB did not significantly improve working memory (k = 4, Hedges’ g = 0.388, 95% CI = −0.077 to 0.853, *p*-value = 0.102, I^2^ = 0) nor episodic memory (k = 2, Hedges’ g = 0.386, 95% CI = −0.454 to 1.225, *p*-value = 0.368, I^2^ = 40.066).

#### 3.4.5. SMR Up-Training vs. Alpha Up-Training

SMR up-training protocols improved working memory (k = 5, Hedges’ g = 0.710, 95% CI = 0.483 to 0.937, *p*-value < 0.001, I^2^ = 0) and episodic memory (k = 6, Hedges’ g = 0.731, 95% CI = 0.294 to 1.169, *p*-value = 0.001, I^2^ = 72.652) in the elderly. Alpha up-training improved working memory (k = 3, Hedges’ g = 0.721, 95% CI = 0.232 to 1.209, *p*-value = 0.004, I^2^ = 0) but did not achieve significance for episodic memory (k = 3, Hedges’ g = 0.339, 95% CI = −0.009 to 0.687, *p*-value = 0.056, I^2^ = 0) in the elderly.

### 3.5. Meta-Regression

Table 3 shows the results of the meta-regression analyses. The total number of sessions, minutes per session, and total training time were not significant moderators for the effect of NFB on either working memory or episodic memory function in the elderly. Studies with an older mean age of participants were associated with slightly less effectiveness of NFB on episodic memory (k = 12, slope = −0.069, *p*-value = 0.006), but the mean age of the participants in the studies did not significantly influence working memory (k = 11, slope = 0.029, *p*-value = 0.221).

## 4. Discussion

In this study, we assessed the effectiveness of NFB on working and episodic memory in the elderly. Our findings indicate that NFB improved both types of memory in the general elderly population and in those with MCI. Sufficient training time (>300 min) was important for achieving these benefits. Regarding protocols, SMR up-training enhanced both types of memory, while alpha up-training only improved working memory. Older age was associated with less NFB effectiveness on episodic but not working memory. In summary, NFB can improve key memories in the elderly, particularly with adequate training time and appropriate protocols. Older age may limit the benefits for episodic memory.

Across subgroups, NFB consistently improved working memory with a moderate effect size and low heterogeneity. In contrast, effects on episodic memory were more variable. Episodic memory is complex, involving encoding, consolidation, and retrieval across multiple brain regions [31]. We suggest that training one EEG band or region may, therefore, insufficiently target all aspects. On the other hand, working memory represents a simpler temporary information storage closely linked to frontal lobe function [32]. Most training protocols in our included studies targeted the frontal–parietal region (12/14 studies). Meta-regression showed advanced age only reduced NFB efficacy for episodic but not working memory. Compared to training one location and rhythm, episodic memory benefits may decrease with overall brain aging. However, our findings suggest sufficient training time is key for both types of memory, regardless of age. In summary, even though episodic memory gains may lessen with age, adequate NFB can still improve this complex faculty in the elderly, alongside more robust working memory benefits.

Compared to young people, the elderly showed an increase in low-frequency EEG bands such as delta and theta and a decrease in the high-alpha band [33]. A decrease in the alpha band and an increase in the theta band were also found in the elderly with Alzheimer’s disease compared to the normal elderly, and the EEG changes in patients with MCI fell in between those of individuals with Alzheimer’s disease and normal elderly individuals [34]. Therefore, a higher theta band and weaker alpha band might be associated with brain aging and degeneration. In addition to the alpha rhythm, low-beta rhythm is associated with memory formation [35]. Notably, SMR (ranged from high-alpha to low-beta band) up-training has been widely applied to enhance attention, which may also benefit memory function. Our findings indicate that NFB improves both working and episodic memory in healthy elderly people and those with MCI, with slightly larger effects in MCI. Notably, most of the NFB studies we included (10/14) employed alpha or SMR up-training, demonstrating memory benefits even though alpha training only trended toward improving episodic memory. NFB training shows short-term enhancement of memory function in the elderly, though long-term impacts require further study. The durability and consistency of effects on broader cognition also warrant additional research. Nonetheless, NFB demonstrates potential for dementia prevention and rehabilitation. Broader research and application of optimized NFB protocols are warranted to confirm and extend these preliminary benefits on memory and cognition in aging.

Using cognitive function tests as the outcome measure in research is relatively less prone to the placebo effect. Conversely, there is a possibility of the emergence of learning effects. The learning effect may diminish as the interval between assessments increases. Only one included study had an 8-day interval between assessments [28], while the rest ranged from over 3 weeks to as long as 12 weeks. The control arm of the study by Reis et al. did not show a significant effect on working memory function within the group analysis, but the NFB arm did [28]. Although in the subgroup analysis of controlled studies vs. pre–post studies, the effect sizes in the pre–post studies group are slightly larger than those in the controlled studies group, the difference did not reach statistical significance, both in working memory and episodic memory. Notably, episodic memory can be divided into verbal and non-verbal (visuospatial) memory, each associated with distinct anatomical substrates [36]. The studies we included in our analysis focused on verbal memory as their primary outcome. In interpreting our findings regarding episodic memory, it is important to acknowledge this distinction. Future studies are warranted to validate the effectiveness of NFB on non-verbal episodic memory. On the other hand, the measurement tools used in the included studies varied, which might contribute to heterogeneity. Different measurement tools have varying test contents, test durations, and scaling scores, and there may also be different learning effects due to varying intervals between pre- and post-assessment and different tools. At least in the primary analysis of working memory, the overall heterogeneity is low (I^2^ = 0), indicating a certain degree of reliability in the effectiveness of NFB on working memory. In episodic memory, although moderate heterogeneity was observed in the primary analysis presentation, partial resolution was also achieved in subgroup analyses. Perhaps in future NFB trials, there could be consensus and uniformity in measurement tools and protocols, allowing for a clearer assessment of the effectiveness of NFB.

### Limitations

This study still has several limitations. First, most of the clinical trials we included had open-label or single-blind designs with relatively small sample sizes, which might be associated with a risk of bias. Future double-blinded randomized controlled trials of NFB with large sample sizes are needed to strengthen our findings. Secondly, we did not perform a meta-regression analysis for education level and female percentage. Few studies included in our analysis provided data on the education level of participants, and most of the included studies were small. Gender differences are relatively non-representative. Thirdly, we did not analyze data on post-NFB EEG changes because the format of EEG data provided by each study varied. If we could simultaneously analyze changes in EEG signals before and after NFB along with alterations in memory function, it would undoubtedly provide a clearer understanding of the mechanisms and effects of NFB. Finally, the study results only reflect memory function immediately post-intervention, and the long-term sustainability of the memory function enhancement effects remains unknown.

## 5. Conclusions

This meta-analysis, comprising 14 clinical trials, found that NFB might improve working memory and episodic memory in the elderly, both healthy elderly individuals and those with MCI. Adequate training time (more than 300 min) was necessary to achieve significant effects. NFB training has demonstrated potential in dementia prevention or rehabilitation. Early intervention for MCI and a combination of NFB training with other cognitive interventions, such as cognitive training exercise, physical exercise, and dietary intervention, might further help slow down the progression of cognitive decline and improve overall brain function. Assessing the long-term and regular effects of NFB training on memory function and other cognitive domains in the elderly is essential for further application.

## Figures and Tables

**Table 1:** The characteristics and demographics of the included studies.

Study Name	Design	Clinical Condition	EEG Band/Controls	Sample (*n*)/Age (Years)	Sessions/Minutes per Session	Outcome of Interest	
						WM	EM
Alatorre–Cruz 2022 [20]	SBRCT	MCI	Theta (−)	10/67.5	30/30	WAIS-III-WMI	NEUROPSI—recall
			Sham	8/68.6			
Becerra 2012 [11]	OLRCT	MCI	Theta (−)	7/65.8	30/30	WAIS-III-WMI	NEUROPSI—memory
			Sham	7/67.0			
Campos da Paz 2018 [23]	OLRCT	Elderly	SMR (+)	7/69.1	10/30	DMST	
			Sham	6/69.1			
Gomez-Pilar 2016 [25]	OLRCT	Elderly	SMR (+)	31/68.3	5/NA	Luria—AND immediate memory	Luria—AND logical memory
			No NF	32/68.0			
Jang 2019 [21]	OLPP	MCI	SMR (+)	5/66.5	16/45		CNSVS—composite memory
Lecomte 2011 [26]	OLRCT	Elderly	Alpha (+), alpha/theta ratio (+)	10/75.3	4/60	SMB learning test	SMB recall test
			Relaxation	10/75.3			
Lavy 2021 [12]	SBRCT	MCI	Alpha (+)	15/70.2	12/30	NeuroTrax battery immediate verbal recall	NeuroTrax battery delayed verbal recall
			Sham	15/74.2			
Lavy 2019 [22]	OLPP	MCI	Alpha (+)	11/70	10/30		NeuroTrax battery delayed verbal recall
Marlats 2020 [13]	OLPP	MCI	SMR (+)/theta (−)	32/76.1	20/45	Forward and backward digit span	Logic memory-recall
Marcos-Martínez 2021 [27]	OLRCT	Elderly	SMR (+)	11/69.4	5/90	Luria—AND immediately memory	Luria—AND logical memory
Reis 2016 [28]	OLRCT	Elderly	Alpha (+), theta (+)	9/65.97	8/30	M. Rot	
			Sham	6/65.97			
Staufenbeil 2014 [29]	DBRCT	Elderly	SMR (+)	10/66.4	8/NA		Encoding memory delayed verbal recall
			Gamma (+)	10/69.2			
van Eijk 2017 [24]	OLRCT	Elderly	SMR (+)	10/77.9	10/21	RAVLT immediate recall	RAVLT delayed recall
			No NF	6/79.2			
Wang 2013 [30]	OLRCT	Elderly	Theta (+)	8/65.0	12/15	Sternberg word recognition task	
			Sham	8/64.6			

SBRCT, single-blind randomized controlled trials; OLPP, open-label pre–post; OLRCT, open-label randomized controlled trials; OLCT, open-label controlled trial; DBRCT, double-blind randomized controlled trials; MCI, mild cognitive impairment; WM, working memory; EM, episodic memory; SMR, sensorimotor rhythm; WAIS-III-WMI, Wechsler adult intelligence scale, third version, working memory index; CNSVS, central nervous system vital signs; DMST, delayed matched to sample task; Luria—AND, Luria adult neuropsychological diagnosis; SMB, Signoret memory battery; M. Rot, the matrix rotation test; RAVLT, the Rey auditory verbal learning test.

**Table 2:** Subgroup analyses of neurofeedback on working memory and episodic memory.

Controlled Studies vs. Single Arm Pre–Post Studies							
	k	Hedge’s g	Lower Limit	Upper Limit	*p*-Value for Effect Size	I^2^	*p*-Value for Heterogeneity
WM–CT	9	0.548	0.277	0.818	<0.001	0	0.973
WM–PP	2	0.765	0.436	1.093	<0.001	19.662	0.265
EM–CT	6	0.539	0.170	0.909	0.004	24.664	0.249
EM–PP	6	0.662	0.282	1.042	0.001	70.938	0.004
MCI vs. Healthy elderly people							
WM–MCI	4	0.812	0.549	1.074	<0.001	0	0.510
WM–HE	7	0.495	0.213	0.778	0.001	0	0.993
EM–MCI	6	0.503	0.088	0.919	0.018	61.028	0.022
EM–HE	6	0.729	0.483	0.976	<0.001	0	0.472
≥10 sessions vs. <10 sessions							
WM–≥10	7	0.728	0.491	0.966	<0.001	0	0.603
WM–<10	4	0.546	0.219	0.873	0.001	0	0.951
EM–≥10	7	0.534	0.148	0.920	0.007	57.847	0.027
EM–<10	5	0.716	0.445	0.986	<0.001	10.813	0.344
≥300 total minutes vs. <300 total minutes							
WM–≥300	6	0.743	0.510	0.976	<0.001	0	0.612
WM–<300	4	0.388	−0.077	0.853	0.102	0	0.962
EM–≥300	7	0.516	0.156	0.876	0.005	57.381	0.027
EM–<300	2	0.386	−0.454	1.225	0.368	40.066	0.196
SMR vs. alpha vs. others							
WM–SMR	5	0.710	0.483	0.937	<0.001	0	0.624
WM–alpha	3	0.721	0.232	1.209	0.004	0	0.475
WM–others	3	0.346	−0.195	0.887	0.210	0	0.981
EM–SMR	6	0.731	0.294	1.169	0.001	72.652	0.003
EM–alpha	3	0.339	−0.009	0.687	0.056	0	0.383
EM–others	3	0.802	0.379	1.225	<0.001	0	0.561

WM, working memory; EM, episodic memory; CT, controlled trials; PP, pre–post trials; MCI, mild cognitive impairment; HE, healthy elderly; SMR, Sensorimotor rhythm.

**Table 3:** Meta-regression of neurofeedback on working memory and episodic memory.

	k	Coefficient	Standard Error	Lower Limit	Upper Limit	*p*-Value
Mean age–study level						
WM	11	0.029	0.024	−0.017	0.075	0.221
EM	12	−0.069	0.025	−0.118	−0.019	0.006
Total sessions						
WM	11	0.008	0.012	−0.016	0.032	0.514
EM	12	−0.009	0.017	−0.041	0.023	0.581
Minutes/sessions						
WM	10	−0.000	0.005	−0.011	0.010	0.938
EM	9	−0.000	0.008	−0.016	0.016	0.987
Total training time						
WM	10	0.000	0.000	−0.000	0.001	0.223
EM	9	0.000	0.000	−0.001	0.001	0.896

WM, working memory; EM, episodic memory.

## Data Availability

Data are contained within the article and Supplementary Materials.

## Supplementary Materials

The following supporting information can be downloaded at https://www.mdpi.com/article/10.3390/medicina60030369/s1. Supplement S1. Checklist of PRISMA guidelines; Supplement S2. Search strategy; Supplement S3. Reasons for exclusion; Supplement S4. Quality assessment across all studies with JADAD score.
