# Supplementary material for: Effectiveness of Electroencephalography Neurofeedback for Improving Working Memory and Episodic Memory in the Elderly: A Meta-Analysis
Source: Medicina (Kaunas). 2024 Feb 22;60(3):369. doi: 10.3390/medicina60030369 (PMC10972127; doi:10.3390/medicina60030369)
Supplement: Supplementary file 1 [file medicina-60-00369-s001.zip › medicina-2861222-supplementary.pdf]

Supplement S1. Checklist of PRISMA guideline

Section and Topic	Item #	Checklist item	Location where item is reported
**TITLE**			
Title	1	Identify the report as a systematic review.	title
**ABSTRACT**			
Abstract	2	See the PRISMA 2020 for Abstracts checklist.	Abstract section
**INTRODUCTION**			
Rationale	3	Describe the rationale for the review in the context of existing knowledge.	1st -3rd paragraph of introduction
Objectives	4	Provide an explicit statement of the objective(s) or question(s) the review addresses.	4th paragraph of introduction
**METHODS**			
Eligibility criteria	5	Specify the inclusion and exclusion criteria for the review and how studies were grouped for the syntheses.	paragraph of " Inclusion and exclusion criteria" of methods
Information sources	6	Specify all databases, registers, websites, organisations, reference lists and other sources searched or consulted to identify studies. Specify the date when each source was last searched or consulted.	paragraph of " search strategy " of methods<br>appendix 2
Search strategy	7	Present the full search strategies for all databases, registers and websites, including any filters and limits used.	paragraph of "search strategy" of methods<br>supplement 2
Selection process	8	Specify the methods used to decide whether a study met the inclusion criteria of the review, including how many reviewers screened each record and each report retrieved, whether they worked independently, and if applicable, details of automation tools used in the process.	paragraph of "search strategy" of methods<br>Figure 1

Section and Topic	Item #	Checklist item	Location where item is reported
Data collection process	9	Specify the methods used to collect data from reports, including how many reviewers collected data from each report, whether they worked independently, any processes for obtaining or confirming data from study investigators, and if applicable, details of automation tools used in the process.	paragraph of " Data extraction and Outcome Definition" of methods
Data items	10a	List and define all outcomes for which data were sought. Specify whether all results that were compatible with each outcome domain in each study were sought (e.g. for all measures, time points, analyses), and if not, the methods used to decide which results to collect.	paragraph of " Data extraction and Outcome Definition" of methods
	10b	List and define all other variables for which data were sought (e.g. participant and intervention characteristics, funding sources). Describe any assumptions made about any missing or unclear information.	paragraph of " Data extraction and Outcome Definition" of methods
Study risk of bias assessment	11	Specify the methods used to assess risk of bias in the included studies, including details of the tool(s) used, how many reviewers assessed each study and whether they worked independently, and if applicable, details of automation tools used in the process.	paragraph of " Assessment of bias" of methods
Effect measures	12	Specify for each outcome the effect measure(s) (e.g. risk ratio, mean difference) used in the synthesis or presentation of results.	paragraph of " Data analyses" of methods
Synthesis methods	13a	Describe the processes used to decide which studies were eligible for each synthesis (e.g. tabulating the study intervention characteristics and comparing against the planned groups for each synthesis (item #5)).	paragraph of " Data analyses" of methods
	13b	Describe any methods required to prepare the data for presentation or synthesis, such as handling of missing summary statistics, or data conversions.	paragraph of " Data analyses" of methods

Section and Topic	Item #	Checklist item	Location where item is reported
	13c	Describe any methods used to tabulate or visually display results of individual studies and syntheses.	paragraph of " Data analyses" of methods
	13d	Describe any methods used to synthesize results and provide a rationale for the choice(s). If meta-analysis was performed, describe the model(s), method(s) to identify the presence and extent of statistical heterogeneity, and software package(s) used.	paragraph of " Data analyses" of methods
	13e	Describe any methods used to explore possible causes of heterogeneity among study results (e.g. subgroup analysis, meta-regression).	paragraph of " Data analyses" of methods
	13f	Describe any sensitivity analyses conducted to assess robustness of the synthesized results.	paragraph of " Data analyses" of methods
Reporting bias assessment	14	Describe any methods used to assess risk of bias due to missing results in a synthesis (arising from reporting biases).	Not applicable
Certainty assessment	15	Describe any methods used to assess certainty (or confidence) in the body of evidence for an outcome.	Not applicable
**RESULTS**			
Study selection	16a	Describe the results of the search and selection process, from the number of records identified in the search to the number of studies included in the review, ideally using a flow diagram.	Figure 1 (PRISMA 2020 flow diagram)
	16b	Cite studies that might appear to meet the inclusion criteria, but which were excluded, and explain why they were excluded.	Supplement S2, S3
Study characteristics	17	Cite each included study and present its characteristics.	Table 1
Risk of bias in	18	Present assessments of risk of bias for each included study.	paragraph of " Methodology quality of the

Section and Topic	Item #	Checklist item	Location where item is reported
studies			included studies" of result<br>Supplement S4
Results of individual studies	19	For all outcomes, present, for each study: (a) summary statistics for each group (where appropriate) and (b) an effect estimate and its precision (e.g. confidence/credible interval), ideally using structured tables or plots.	paragraph of " Co-primary outcomes; working memory and episodic memory" of result<br>Figure 2
Results of syntheses	20a	For each synthesis, briefly summarise the characteristics and risk of bias among contributing studies.	paragraph of " Co-primary outcomes; working memory and episodic memory" of result<br>Figure 2
	20b	Present results of all statistical syntheses conducted. If meta-analysis was done, present for each the summary estimate and its precision (e.g. confidence/credible interval) and measures of statistical heterogeneity. If comparing groups, describe the direction of the effect.	paragraph of " Co-primary outcomes; working memory and episodic memory" of result<br>Figure 2
	20c	Present results of all investigations of possible causes of heterogeneity among study results.	paragraph of " Subgroup analyses" of result<br>Table 2<br>paragraph of " Meta-regression" of result<br>Table 3
	20d	Present results of all sensitivity analyses conducted to assess the robustness of the synthesized results.	paragraph of " Subgroup analyses" of result<br>Table 2

Section and Topic	Item #	Checklist item	Location where item is reported
			paragraph of " Meta-regression" of result<br>Table 3
Reporting biases	21	Present assessments of risk of bias due to missing results (arising from reporting biases) for each synthesis assessed.	eFigure 2, 3<br>paragraph of "Quality of the included studies" of result
Certainty of evidence	22	Present assessments of certainty (or confidence) in the body of evidence for each outcome assessed.	Not applicable
**DISCUSSION**			
Discussion	23a	Provide a general interpretation of the results in the context of other evidence.	1st paragraph of discussion
	23b	Discuss any limitations of the evidence included in the review.	5th paragraph of discussion
	23c	Discuss any limitations of the review processes used.	5th paragraph of discussion
	23d	Discuss implications of the results for practice, policy, and future research.	Conclusions section
**OTHER INFORMATION**			
Registration and protocol	24a	Provide registration information for the review, including register name and registration number, or state that the review was not registered.	OSF(https://osf.io/q94k5/)
	24b	Indicate where the review protocol can be accessed, or state that a protocol was not prepared.	OSF(https://osf.io/q94k5/)
	24c	Describe and explain any amendments to information provided at registration or in the protocol.	1. We did not use Medline database, because it is similar to Pubmed<br>2. We additionally performed meta-regression for mean age and

Section and Topic	Item #	Checklist item	Location where item is reported
			treatment duration
Support	25	Describe sources of financial or non-financial support for the review, and the role of the funders or sponsors in the review.	None
Competing interests	26	Declare any competing interests of review authors.	None
Availability of data, code and other materials	27	Report which of the following are publicly available and where they can be found: template data collection forms; data extracted from included studies; data used for all analyses; analytic code; any other materials used in the review.	Not applicable

Supplement S2. Search strategy

Pubmed: (EEG OR electroencephalograph*) AND (neurofeedback OR biofeedback) AND (memor* OR cogniti*) AND (elderly OR old OR older OR MCI OR mild cognitive impairment) with restriction to flitters of clinical trials (34)

Web of Science: (EEG OR electroencephalograph*) AND (neurofeedback OR biofeedback) AND (memor* OR cogniti*) AND (elderly OR old OR older OR MCI OR mild cognitive impairment) (74)

Embase: (EEG OR electroencephalograph*) AND (neurofeedback OR biofeedback) AND (memor* OR cogniti*) AND (elderly OR old OR older OR MCI OR mild cognitive impairment) with restriction to flitters of clinical articles (75)

Cochrane Central Register of Controlled Trials (CENTRAL): (EEG OR electroencephalograph*) AND (neurofeedback OR biofeedback) AND (memor* OR cogniti*) AND (elderly OR old OR older OR MCI OR mild cognitive impairment) (39)

Clinical trials gov: (EEG OR electroencephalograph*) AND (neurofeedback OR biofeedback) AND (memor* OR cogniti*) AND (elderly OR old OR older OR MCI OR mild cognitive impairment) (6)

Supplement S3. Reasons for exclusion

Outcome not interest (n=19)

1. Arruda JE, Amoss RT, Coburn KL, McGee H. A quantitative electroencephalographic correlate of sustained attention processing. *Applied Psychophysiology and Biofeedback*. Mar 2007;32(1):11-17. doi:10.1007/s10484-007-9030-1
2. Asai T, Hamamoto T, Kashihara S, Imamizu H. Real-Time Detection and Feedback of Canonical Electroencephalogram Microstates: Validating a Neurofeedback System as a Function of Delay. *Frontiers in Systems Neuroscience*. Feb 2022;16786200. doi:10.3389/fnsys.2022.786200
3. Barth B, Mayer-Carius K, Strehl U, et al. A randomized-controlled neurofeedback trial in adult attention-deficit/hyperactivity disorder. *Sci Rep*. Aug 19 2021;11(1):16873. doi:10.1038/s41598-021-95928-1
4. Bazanova OM, Kholodina N, Nikolenko ED. Neurofeedback training efficiency of regularly cycling women may differ throughout the month, depending on progesterone status. Journal article; Conference proceeding. *International journal of psychophysiology*. 2014;94(2):148-149. doi:10.1016/j.ijpsycho.2014.08.670
5. Bielas J, Michalczyk L. Beta Neurofeedback Training Improves Attentional Control in the Elderly. *Psychological Reports*. Feb 2021;124(1):54-69. 0033294119900348. doi:10.1177/0033294119900348
6. Hohenfeld C, Kuhn H, Müller C, et al. Changes in brain activation related to visuo-spatial memory after real-time fMRI neurofeedback training in healthy elderly and Alzheimer's disease. Article. *Behavioural Brain Research*. 2020;381doi:10.1016/j.bbr.2019.112435
7. Hosseini Z, Delpazirian R, Lanjanian H, Salarifar M, Hassani-Abharian P. Computer Gaming and Physiological Changes in the Brain: An Insight from QEEG Complexity Analysis. Article. *Applied psychophysiology and biofeedback*. 2021;46(3):301-308. doi:10.1007/s10484-021-09518-y
8. Hsiao YT, Wu CT, Tsai CF, Liu YH, Trinh TT, Lee CY. EEG-Based Classification Between Individuals with Mild Cognitive Impairment and Healthy Controls Using Conformal Kernel-Based Fuzzy Support Vector Machine. *International Journal of Fuzzy Systems*. Nov 2021;23(8):2432-2448. doi:10.1007/s40815-021-01186-8
9. Israsena P, Jirayucharoensak S, Hemrungrojn S, Pan-Ngum S. Brain Exercising Games With Consumer-Grade Single-Channel Electroencephalogram Neurofeedback: Pre-Post Intervention Study. *Jmir Serious Games*. Apr-Jun 2021;9(2)e26872. doi:10.2196/26872
10. Jaeger M, Mirkovic B, Bleichner MG, Debener S. Decoding the Attended Speaker From EEG Using Adaptive Evaluation Intervals Captures Fluctuations in Attentional Listening. *Frontiers in Neuroscience*. Jun 2020;14603. doi:10.3389/fnins.2020.00603
11. Kim SO, Pyun SB, Park SA. Improved Cognitive Function and Emotional Condition Measured Using Electroencephalography in the Elderly during Horticultural Activities. *Hortscience*. Aug 2021;56(8):985-994. doi:10.21273/hortsci15818-21
12. Li X, Zhang J, Li XD, Cui W, Su R. Neurofeedback Training for Brain Functional Connectivity Improvement in Mild Cognitive Impairment. Article. *Journal of Medical and Biological Engineering*. 2020;40(4):484-495. doi:10.1007/s40846-020-00531-w
13. Meekes J, Debener S, Zich C, Bleichner MG, Kranczioch C. Does Fractional Anisotropy Predict Motor

Imagery Neurofeedback Performance in Healthy Older Adults? *Frontiers in Human Neuroscience*. Feb 2019;1369. doi:10.3389/fnhum.2019.00069
14. Ros T, Baars BJ, Lanius RA, Vuilleumier P. Tuning pathological brain oscillations with neurofeedback: a systems neuroscience framework. *Frontiers in Human Neuroscience*. Dec 2014;81008. doi:10.3389/fnhum.2014.01008
15. Su R, Li X, Liu Y, Cui W, Xie P, Han Y. Evaluation of the Brain Function State During Mild Cognitive Impairment Based on Weighted Multiple Multiscale Entropy. Article. *Frontiers in Aging Neuroscience*. 2021;13doi:10.3389/fnagi.2021.625081
16. Tang HY, Vitiello MV, Perlis M, Riegel B. Open-Loop Neurofeedback Audiovisual Stimulation: A Pilot Study of Its Potential for Sleep Induction in Older Adults. *Applied Psychophysiology and Biofeedback*. Sep 2015;40(3):183-188. doi:10.1007/s10484-015-9285-x
17. Weon H, Yoo J, Yu J, Park M, Son H. Effects of cognicise-neurofeedback on health locus of control, depression, and quantitative electroencephalography alpha asymmetry in elderly women. Journal article. *Brain sciences*. 2021;11(7)doi:10.3390/brainsci11070899
18. Zich C, Debener S, De Vos M, Frerichs S, Maurer S, Kranczioch C. Lateralization patterns of covert but not overt movements change with age: An EEG neurofeedback study. *Neuroimage*. Aug 2015;116:80-91. doi:10.1016/j.neuroimage.2015.05.009
19. Zich C, Debener S, Thoene AK, Chen LC, Kranczioch C. Simultaneous EEG-fNIRS reveals how age and feedback affect motor imagery signatures. *Neurobiology of Aging*. Jan 2017;49:183-197. doi:10.1016/j.neurobiolaging.2016.10.011

Other populations (n=29)

1. Bezmaternykh D, Melnikov M, Savelov A, Petrovskiy E, Sokhadze E, Shtark M. Real-Time fMRI-EEG Neurofeedback for Stroke Rehabilitation. Conference Abstract. *NeuroRegulation*. 2022;9(4):201. doi:10.15540/nr.9.4.198
2. Bialkowska J, Mroczkowska D, Wojtkiewicz J, Maksymowicz W. The use of EEG biofeedback in rehabilitation of a patient with amyotrophic lateral sclerosis with cognitive, mood and motivation disorders. Article. *Polish Annals of Medicine*. 2018;25(1):68-73. doi:10.29089/2017.17.00012
3. Chen TC, Lin IM. The learning effects and curves during high beta down-training neurofeedback for patients with major depressive disorder. Article. *Journal of Affective Disorders*. 2020;266:235-242. doi:10.1016/j.jad.2020.01.175
4. Choi SW, Chi SE, Chung SY, Kim JW, Ahn CY, Kim HT. Is Alpha Wave Neurofeedback Effective with Randomized Clinical Trials in Depression? A Pilot Study. *Neuropsychobiology*. 2011;63(1):43-51. doi:10.1159/000322290
5. Cortoos A, De Valck E, Arns M, Breteler MH, Cluydts R. An exploratory study on the effects of tele-neurofeedback and tele-biofeedback on objective and subjective sleep in patients with primary insomnia. *Appl Psychophysiol Biofeedback*. Jun 2010;35(2):125-34. doi:10.1007/s10484-009-9116-z
6. Escolano C, Navarro-Gil M, Garcia-Campayo J, Minguez J. EEG-based upper-alpha neurofeedback for cognitive enhancement in major depressive disorder: a preliminary, uncontrolled study. *Annu Int Conf IEEE*

Eng Med Biol Soc. 2013;2013:6293-6. doi:10.1109/embc.2013.6610992
7. Fink M, Pasche S, Schmidt K, et al. Neurofeedback Treatment Affects Affective Symptoms, But Not Perceived Cognitive Impairment in Cancer Patients: Results of an Explorative Randomized Controlled Trial. Article. *Integrative Cancer Therapies*. 2023;22doi:10.1177/15347354221149950
8. Galvin-McLaughlin D, Klee D, Memmott T, et al. Methodology and preliminary data on feasibility of a neurofeedback protocol to improve visual attention to letters in mild Alzheimer's disease. Article. *Contemporary Clinical Trials Communications*. 2022;28doi:10.1016/j.conctc.2022.100950
9. Grosselin F, Breton A, Yahia-Cherif L, et al. Alpha activity neuromodulation induced by individual alpha-based neurofeedback learning in ecological context: a double-blind randomized study. *Sci Rep*. Sep 16 2021;11(1):18489. doi:10.1038/s41598-021-96893-5
10. Ivanova GE, Bushkova YV, Suvorov AY, et al. Use of a BCI-Exoskeleton Simulator with Multichannel Biofeedback in a Multidisciplinary Rehabilitation Program in Poststroke Patients. Article. *Neuroscience and Behavioral Physiology*. 2018;48(9):1100-1105. doi:10.1007/s11055-018-0673-6
11. Keune PM, Hansen S, Sauder T, et al. Frontal brain activity and cognitive processing speed in multiple sclerosis: An exploration of EEG neurofeedback training. *Neuroimage Clin*. 2019;22:101716. doi:10.1016/j.nicl.2019.101716
12. Kober SE, Schweiger D, Reichert JL, Neuper C, Wood G. Upper Alpha Based Neurofeedback Training in Chronic Stroke: Brain Plasticity Processes and Cognitive Effects. *Applied Psychophysiology and Biofeedback*. Mar 2017;42(1):69-83. doi:10.1007/s10484-017-9353-5
13. Kober SE, Schweiger D, Witte M, et al. Specific effects of EEG based neurofeedback training on memory functions in post-stroke victims. Article. *Journal of NeuroEngineering and Rehabilitation*. 2015;12(1)doi:10.1186/s12984-015-0105-6
14. Kovyazina M, Trofimova A, Isaychev S, Chernorizov A. Neurofeedback in psychological rehabilitation of stroke patients. Conference Abstract. *Cerebrovascular Diseases*. 2018;45:339. doi:10.1159/000490132
15. Kovyazina M, Varako N, Rasskazova E, Trofimova A, Dobrushina O. Overcoming stroke-related disability through rehabilitation: the usefulness of neuropsysiological methods. Conference Abstract. *European Psychiatry*. 2019;56:S237-S238. doi:10.1016/j.eurpsy.2019.01.003
16. Kwan Y, Yoon S, Suh S, Choi S. A Randomized Controlled Trial Comparing Neurofeedback and Cognitive-Behavioral Therapy for Insomnia Patients: Pilot Study. *Appl Psychophysiol Biofeedback*. Jun 2022;47(2):95-106. doi:10.1007/s10484-022-09534-6
17. Luctkar-Flude M, Groll D, Tyerman J. Using neurofeedback to manage long-term symptoms in cancer survivors: Results of a survey of neurofeedback providers. Article. *European Journal of Integrative Medicine*. 2017;12:172-176. doi:10.1016/j.eujim.2017.06.003
18. Luijmes RE, Pouwels S, Boonman J. The effectiveness of neurofeedback on cognitive functioning in patients with Alzheimer's disease: Preliminary results. Article. *Neurophysiologie Clinique*. 2016;46(3):179-187. doi:10.1016/j.neucli.2016.05.069
19. Mosanezhad-Jeddi E, Nazari MA. Effectiveness of EEG-biofeedback on attentiveness, working memory and quantitative electroencephalography on reading disorder. Article. *Iranian Journal of Psychiatry and Behavioral Sciences*. 2013;7(2):35-43.
20. Pei G, Wang S, Shi Z, et al. Neurofeedback technique based on mismatched negativity in improvement

of cognitive function. Article. *Chinese Journal of Neuromedicine*. 2020;19(4):330-336. doi:10.3760/cma.j.cn115354-20200216-00081
21. Scott WC, Kaiser D, Othmer S, Sideroff SI. Effects of an EEG biofeedback protocol on a mixed substance abusing population. *Am J Drug Alcohol Abuse*. 2005;31(3):455-69. doi:10.1081/ada-200056807
22. Singh F, Shu IW, Hsu SH, Link P, Pineda JA, Granholm E. Modulation of frontal gamma oscillations improves working memory in schizophrenia. Article. *NeuroImage: Clinical*. 2020;27doi:10.1016/j.nicl.2020.102339
23. Surmeli T, Eralp E, Mustafazade I, Kos H, Özer GE, Surmeli OH. Quantitative EEG Neurometric Analysis-Guided Neurofeedback Treatment in Dementia: 20 Cases. How Neurometric Analysis Is Important for the Treatment of Dementia and as a Biomarker? Article. *Clinical EEG and Neuroscience*. 2016;47(2):118-133. doi:10.1177/1550059415590750
24. Surmeli T, Ertem A. Post WISC-R and TOVA improvement with QEEG guided neurofeedback training in mentally retarded: A clinical case series of behavioral problems. Article. *Clinical EEG and Neuroscience*. 2010;41(1):32-41. doi:10.1177/155005941004100108
25. Takabatake K, Kunii N, Nakatomi H, et al. Musical Auditory Alpha Wave Neurofeedback: validation and Cognitive Perspectives. Journal article. *Applied psychophysiology and biofeedback*. 2021;46(4):323-334. doi:10.1007/s10484-021-09507-1
26. Thornton KE. The improvement/rehabilitation of auditory memory functioning with EEG biofeedback. Article. *NeuroRehabilitation*. 2002;17(1):69-80. doi:10.3233/nre-2002-17109
27. Wang JX, Wang WQ, Hou ZG. EEG-Based Focus of Attention Tracking and Regulation During Dual-Task Training for Neural Rehabilitation of Stroke Patients. *Ieee Transactions on Biomedical Engineering*. Mar 2023;70(3):920-930. doi:10.1109/tbme.2022.3205066
28. Xia MY, Xu PF, Yang YB, et al. Frontoparietal Connectivity Neurofeedback Training for Promotion of Working Memory: An fNIRS Study in Healthy Male Participants. *Ieee Access*. 2021;9:62316-62331. doi:10.1109/access.2021.3074220
29. Yuan Z, Peng Y, Wang L, et al. Effect of BCI-Controlled Pedaling Training System with Multiple Modalities of Feedback on Motor and Cognitive Function Rehabilitation of Early Subacute Stroke Patients. Article. *IEEE Transactions on Neural Systems and Rehabilitation Engineering*. 2021;29:2569-2577. doi:10.1109/TNSRE.2021.3132944

Review, Case report, Protocols, or Conference abstract (n=29)

1. Blanchet S, Chikhi S, Maillard C, Gaston-Bellegarde A, Orriols E, Piolino P. How neurofeedback training targeting Theta or Theta + Gamma improves attentional and memory efficiency in an ecological situation using virtual reality. Journal article; Conference proceeding. *International journal of psychophysiology*. 2023;188:43. doi:10.1016/j.ijpsycho.2023.05.108
2. Campos-Arteaga G, Torres JF, Morales-Torres R, et al. INVESTIGATING THE IMPACT OF EEG SUBJECT-DEPENDENT NEUROFEEDBACK ON DECLARATIVE MEMORY CONSOLIDATION. Journal article; Conference proceeding. *IBRO neuroscience reports*. 2023;15:S821. doi:10.1016/j.ibneur.2023.08.1702

3. Carelli L, Solca F, Faini A, et al. Brain-Computer Interface for Clinical Purposes: Cognitive Assessment and Rehabilitation. *Biomed Research International*. 2017;20171695290. doi:10.1155/2017/1695290
4. Gomez-Pilar J, Corralejo R, Nicolas-Alonso LF, Álvarez D, Hornero R. Assessment of neurofeedback training by means of motor imagery based-BCI for cognitive rehabilitation. *Annu Int Conf IEEE Eng Med Biol Soc*. 2014;2014:3630-3. doi:10.1109/embc.2014.6944409
5. Gonzalez-Lapez M. Psychoneuroendocrinology of aging: Implications for neuroregulation. Conference Abstract. *NeuroRegulation*. 2021;8(4):202-203. doi:10.15540/nr.8.4.198
6. Gonzalez-Lopez M. Neurofeedback in healthy elderly at risk of cognitive decline. Conference Abstract. *NeuroRegulation*. 2020;7(4):166-167. doi:10.15540/nr.7.4.158
7. Gruzelier JH. EEG-neurofeedback for optimising performance. I: A review of cognitive and affective outcome in healthy participants. *Neuroscience and Biobehavioral Reviews*. Jul 2014;44:124-141. doi:10.1016/j.neubiorev.2013.09.015
8. Ioannides AA. Neurofeedback and the Neural Representation of Self: Lessons From Awake State and Sleep. *Frontiers in Human Neuroscience*. Apr 2018;12142. doi:10.3389/fnhum.2018.00142
9. Isrctn. Neurofeedback for improved mental functioning. Trial registry record. *https://trialsearchwhoint/Trial2aspx?TrialID=ISRCTN19822753*. 2019;
10. Jeong JH, Jung C, Kim J, et al. Investigation of combined treatment of acupuncture and neurofeedback for improving cognitive function in mild neurocognitive disorder A randomized, assessor-blind, pilot study. Article. *Medicine (United States)*. 2021;100(37)doi:10.1097/MD.0000000000027218
11. Jiang Y, Abiri R, Zhao XP. Tuning Up the Old Brain with New Tricks: Attention Training via Neurofeedback. *Frontiers in Aging Neuroscience*. Mar 2017;952. doi:10.3389/fnagi.2017.00052
12. Jiang Y, Jessee W, Hoyng S, et al. Sharpening Working Memory With Real-Time Electrophysiological Brain Signals: Which Neurofeedback Paradigms Work? *Frontiers in Aging Neuroscience*. Mar 2022;14780817. doi:10.3389/fnagi.2022.780817
13. jRcts. The development of treatment using the connectivity neurofeedback for patients with affective disorders. Trial registry record. *https://trialsearchwhoint/Trial2aspx?TrialID=JPRN-jRCTs052180169*. 2019;
14. Klink K, Jaun U, Federspiel A, et al. Targeting hippocampal hyperactivity with real-time fMRI neurofeedback: protocol of a single-blind randomized controlled trial in mild cognitive impairment. Article. *BMC Psychiatry*. 2021;21(1)doi:10.1186/s12888-021-03091-8
15. Koberda JL, Hillier DS, Jones B, Moses A, Koberda L. Application of Neurofeedback in General Neurology Practice. Article. *Journal of Neurotherapy*. 2012;16(3):231-234. doi:10.1080/10874208.2012.705770
16. Laborda-Sánchez F, Cansino S. The Effects of Neurofeedback on Aging-Associated Cognitive Decline: A Systematic Review. *Applied Psychophysiology and Biofeedback*. Mar 2021;46(1):1-10. doi:10.1007/s10484-020-09497-6
17. Li L, Gui X, Huang G, et al. Decoded EEG Neurofeedback-Guided Cognitive Reappraisal Training for Emotion Regulation. 2023.
18. Marlats F, Djabelkhir-Jemmi L, Azabou E, Boubaya M, Pouwels S, Rigaud AS. Comparison of effects between SMR/delta-ratio and beta1/theta-ratio neurofeedback training for older adults with Mild Cognitive Impairment: a protocol for a randomized controlled trial. Journal article. *Trials*. 2019;20

19. Nan WY, Dias APB, Rosa AC. Neurofeedback Training for Cognitive and Motor Function Rehabilitation in Chronic Stroke: Two Case Reports. *Frontiers in Neurology*. Jul 2019;10800. doi:10.3389/fneur.2019.00800
20. Nct. iMeditate at Home for Older Adults With Mild Cognitive Impairment and Caregivers. Trial registry record. *https://clinicaltrialsgov/show/NCT04184037*. 2019;
21. Nct. Neurofeedback Impact on Veterans With mTBI. Trial registry record. *https://clinicaltrialsgov/show/NCT04195685*. 2019;
22. Nct. Assessing the Effects of the Muse Meditation System on Cognition and Well-being. Trial registry record. *https://clinicaltrialsgov/show/NCT04790929*. 2021;
23. Nct. Neurofeedback Training For Older Adults. Trial registry record; Clinical trial protocol. *https://clinicaltrialsgov/ct2/show/NCT05936697*. 2023;
24. Nct. Effects of Motor Imagery and Action Observation on Upper Limb Motor Chances and Cognitive Chances in Parkinson's Disease. Trial registry record. *https://clinicaltrialsgov/show/NCT05696925*. 2023;
25. Ribeiro TF, Carriello MA, de Paula EP, Jr., Garcia AC, da Rocha GL, Teive HAG. Clinical applications of neurofeedback based on sensorimotor rhythm: a systematic review and meta-analysis. *Frontiers in Neuroscience*. Nov 2023;171195066. doi:10.3389/fnins.2023.1195066
26. Su R, Li X, Zhang J, Shi C. A novel eeg feature extracting algorithm and its application on mild cognitive impairment neurofeedback training. Conference Abstract. *Basic and Clinical Pharmacology and Toxicology*. 2020;126:39-40.
27. Su R, Li X, Zhang J, Shi CY. A Novel EEG Feature Extracting Algorithm and its Application on Mild Cognitive Impairment Neurofeedback Training. *Basic & Clinical Pharmacology & Toxicology*. Apr 2020;126:39-40.
28. Trambaiolli LR, Cassani R, Mehler DMA, Falk TH. Neurofeedback and the Aging Brain: A Systematic Review of Training Protocols for Dementia and Mild Cognitive Impairment. *Frontiers in Aging Neuroscience*. Jun 2021;13682683. doi:10.3389/fnagi.2021.682683
29. Zomorrodi R, Saltmarche AE, Loheswaran G, Ho KF, Lim L. EEG data for a significantly improved alzheimer's disease case after photobiomodulation treatment. Conference Abstract. *NeuroRegulation*. 2017;4(3-4):167. doi:10.15540/nr.4.3-4.100

Incomplete data reports (n=2)

1. Jirayucharoensak S, Israsena P, Pan-Ngum S, Hemrungrojn S, Maes M. A game-based neurofeedback training system to enhance cognitive performance in healthy elderly subjects and in patients with amnestic mild cognitive impairment. Journal article. *Clinical interventions in aging*. 2019;14:347-360. doi:10.2147/CIA.S189047
2. Angelakis E, Stathopoulou S, Frymiare JL, Green DL, Lubar JF, Kounios J. EEG neurofeedback: a brief overview and an example of peak alpha frequency training for cognitive enhancement in the elderly. Journal article. *Clinical neuropsychologist*. 2007;21(1):110-129. doi:10.1080/13854040600744839

Other interventions (n=5)

1. Lee TS, Quek SY, Goh SJ, et al. A pilot randomized controlled trial using EEG-based brain-computer

interface training for a Chinese-speaking group of healthy elderly. *Clin Interv Aging*. 2015;10:217-27. doi:10.2147/cia.S73955

2. Meeuwsen KD, Groeneveld KM, Walker LA, Mennenga AM, Tittle RK, White EK. Z-score neurofeedback, heart rate variability biofeedback, and brain coaching for older adults with memory concerns. *Restorative Neurology and Neuroscience*. 2021;39(1):9-37. doi:10.3233/rnn-201053
3. Pei G, Yang R, Shi Z, et al. Enhancing Working Memory Based on Mismatch Negativity Neurofeedback in Subjective Cognitive Decline Patients: A Preliminary Study. Front Aging Neurosci. 2020;12:263. doi:10.3389/fnagi.2020.00263
4. Fotuhi, M., et al. "A personalized 12-week "brain fitness program" for improving cognitive function and increasing the volume of hippocampus in elderly with mild cognitive impairment." The journal of prevention of Alzheimer's disease 3.3 (2016): 133-137.
5. Mendoza Laiz, Nuria, et al. "Potential benefits of a cognitive training program in mild cognitive impairment (MCI)." *Restorative neurology and neuroscience* 36.2 (2018): 207-213.

Supplement S4. Quality assessment across all studies with JADAD score

Study	Randomization	Blinding	Withdrawal	Total
Alatorre-Cruz 2022	1	0	1	2
Becerra 2012	1	0	1	2
Campos da paz 2018	1	0	1	2
Gomez-pilar 2016	1	0	1	2
Jang 2019	0	0	1	1
Lavy 2019	0	0	1	1
Lavy 2021	1	0	1	2
Lecomte 2011	0	0	1	1
Marcos-Martínez 2021	1	0	1	2
Marlats 2020	0	0	1	1
Reis 2016	1	0	1	2
Staufenbeil 2014	2	1	1	4
van Eijk 2017	0	0	1	1
Wang 2013	1	0	1	2
